# Novel *O*-Methylglucoside Derivatives of Flavanone in Interaction with Model Membrane and Transferrin

**DOI:** 10.3390/membranes12100978

**Published:** 2022-10-08

**Authors:** Sylwia Cyboran-Mikołajczyk, Dorota Bonarska-Kujawa, Katarzyna Męczarska, Agnieszka Krawczyk-Łebek, Edyta Kostrzewa-Susłow

**Affiliations:** 1 Department of Physics and Biophysics, Wrocław University of Environmental and Life Sciences, Norwida 25, 50-375 Wrocław, Poland; 2 Department of Food Chemistry and Biocatalysis, Wrocław University of Environmental and Life Sciences, Norwida 25, 50-375 Wrocław, Poland

**Keywords:** 6-methylflavanone, RBC, transferrin, POPC, red blood cells lipids, fluidity, membrane properties, toxicity, structure–activity relationship, *O*-methylglucosides

## Abstract

Flavonoids were biotransformed using various microorganisms, in order to obtain new compounds with potentially high biological activity. The aim of this work was to determine and compare the biological activity of four novel 6-methylflavanone *O*-methylglucosides. The tested compounds have the same flavonoid core structure and an attached *O*-methylglucose and hydroxyl group at different positions of ring A or B. The studies on their biological activity were conducted in relation to phosphatidylcholine membrane, erythrocytes and their membrane, and with human transferrin. These studies determined the compounds’ toxicity and their impact on the physical properties of the membranes. Furthermore, the binding ability of the compounds to holo-transferrin was investigated. The obtained results indicate that used compounds bind to erythrocytes, change their shape and decrease osmotic fragility but do not disrupt the membrane structure. Furthermore, the used compounds ordered the area of the polar heads of lipids and increased membrane fluidity. However, the results indicate the binding of these compounds in the hydrophilic region of the membranes, like other flavonoid glycosides. The used flavanones formed complexes with transferrin without inducing conformational changes in the protein’s structure. The relationship between their molecular structure and biological activity was discussed.

## 1. Introduction

Flavonoids are one of the most studied compounds of natural origin due to their wide spectrum of prohealth properties. They show high biological activity, in particular antioxidant activity, as well as anti-inflammatory, antiatherosclerotic, antimutagenic, and anticarcinogenic properties coupled with their capacity to modulate key cellular enzyme functions [1]. Flavanones are one of the subclasses of flavonoids, which possess a basic C15 phenyl-benzopyrone skeleton. The two benzene rings (rings A and B) are connected to each other through three carbon atoms. Flavanones have a saturated bond between C-2 and C-3 on the C ring and this is the only structural difference between them and flavones. One of the representatives of this group is naringenin, which is widely distributed in bergamot, tomatoes, and other fruits. It exhibits a number of beneficial properties, i.e., antioxidant, antitumor, antiviral, antibacterial, anti-inflammatory, antiadipogenic, and cardioprotective effects [2]. In nature, flavonoids are mainly found in the form of aglycones and glycosides with different sugar substituents. Methylated flavonoids occur in a limited number of plants, i.e., *Cistus, Pisonia, and Kaempferia*, and with two common methylation patterns: *C*-methylation and *O*-methylation [3,4,5].

Both glycosylation and methylation alter the physicochemical properties and biological activity of modified molecules in relation to the substrate (aglycone). Glycosylation increases the solubility and stability of the molecule in water, causing higher bioavailability of these compounds from the diet [6]. Due to altered polarity, size, and structure, access to the lipid phase and hydrophobic free radicals is limited [7]. Regarding biological activity, glycosylated flavonoids generally display lower antioxidant, antidiabetic, anti-inflammatory, antibacterial, antifungal, antitumor, anticoagulant, antiplatelet, antidegranulating, antitrypanosomal, immunomodulatory, and antitubercular activity compared to the corresponding aglycones [8]. Their antioxidant activity usually decreases with an increasing number of sugar moieties. However, the position and structure of the attached sugar unit also affect the bioactivity of flavonoids. On the other hand, glycosylation can enhance, i.e., anti-human immunodeficiency virus (anti-HIV), antirotavirus, antistress, antiobesity, antiadipogenic, and antiallergic activity [8]. Methylation increases the hydrophobicity of flavonoids and improves their affinity for the cell membrane. Due to their higher uptake in different cells, methylated flavonoids generally have elevated bioavailability [9]. Furthermore, unlike unmethylated flavonoids, methylated ones cannot be further methylated by in vivo methyltransferases; therefore, only glucuronides and sulfates are detected as their metabolites [10]. The antioxidant activity of methylated flavonoids may be increased or decreased depending on their hydrophobicity, hydrogen donating ability, and planarity. The anticancer activity of methylated flavonoids strictly depends on the structure of the compounds and types of cancer cells. Moreover, the molecular mechanism responsible for this activity is different and more extensive than in the case of unmethylated molecules [9]. *O*-Methylation is suggested to be an efficient detoxification pathway for flavonoids [11].

Red blood cells (RBCs) represent the main cells in the body’s circulatory system, and their main function is to transport gasses and nutrients in the human body. Erythrocytes are constantly exposed to the action of free radicals while transporting oxygen and carbon dioxide to and from the tissues. Their unique shape and composition cause the role of the erythrocytes to be critical in investigating many disease processes of body systems [12]. The plasma membrane of RBCs is a two-dimensional, multi-component structure composed of a cytoskeleton and a lipid bilayer that is responsible for cell morphology, elasticity, flexibility, and deformability [13]. The interaction between the membrane components keeps RBCs’ structural integrity and original shape and protects them from the force of circulation during their passage into the microcirculation. Alteration of the membrane structure upon exposure to xenobiotics could lead to a release of intracellular components, particularly hemoglobin. Therefore, the morphological changes and the release of hemoglobin help to evaluate the cytotoxic effects of various molecules [12]. Furthermore, the proper functioning of the RBCs depends on the physical properties of their lipid membrane, in particular on membrane fluidity, which determines the activity of membrane proteins. It is well known that membrane fluidity generally depends on the type of phospholipids, the acyl chain length, the degree of fatty acid saturation, and the presence of free or esterified cholesterol. Membrane fluidity is the reciprocal of membrane microviscosity, which in turn is inversely proportional to rotational and lateral diffusion rates of membrane components [14]. It may be changed upon exposure to xenobiotics that bind to the membrane and/or as a consequence of oxidative stress that leads to lipid peroxidation. As a consequence, the conformation of proteins and their cross-linking may be altered, leading to abnormal cell morphology and hemolysis that could disturb the microcirculation [12]. Therefore, the studies of the impact of different natural or synthetic molecules on the physical properties of the lipid membrane allow for the assessment of their biological activity and elucidation of the molecular mechanism responsible for this activity.

Transferrin is the crucial iron-transport glycoprotein in the blood for most life-sustaining processes. It plays a major role in binding and delivering iron into cells with the specific transferrin receptor via a receptor-mediated endocytosis process. It is a single-chain blood plasma protein containing 679 amino acids with a molecular mass of 80 kDa and plasma concentration of about 35 µM, mainly produced in the liver [15]. It occurs in two states: iron-bound holo-transferrin and iron-free apo-transferrin under physiological conditions. Holo-transferrin (HTF) in the “closed conformation” transports two ferric iron into the cells, and then transforms into an apo-transferrin form that possesses an “open conformation” [16,17]. The human serum transferrin is saturated with 30% of iron and exhibits a mixed state of open, closed, and partially open conformations [18]. Transferrin is used to transport and deliver chemotherapeutic drug molecules that can specifically target cancer cells. The transferrin-receptors are located on the cell surfaces, bind to transferrin with high affinity, and then internalize it into the cell. Due to the high demand for iron in tumor cells, for energy production, heme synthesis, and cell proliferation the transferrin-receptors are overexpressed at the surface of cancer cells [19]. After binding to the drug, transferrin delivers it to tumor cells via receptor-mediated endocytosis. Afterwards, the uptake of the drug–transferrin complex into an acidic endosome could result in drug exposure and release. Therefore, the knowledge about the interaction of natural or synthetic molecules with transferrin may help understand the mechanism of xenobiotic–protein interaction in the blood. It may also improve the therapeutic efficiency of cytostatics through the possibility of delivering substances of targeted biological activity to cancer cells that can potentially support their action.

Current research focuses on the search for new compounds of natural origin with high biological activity. For this purpose, the selected active compounds, mainly aglycones, were biotransformed using various microorganisms to obtain new compounds with potentially high biological activity. The aim of this work was to determine and compare the biological activity of four products of 6-methylflavanone fungal biotransformation containing a methyglucose residue in different positions in the structure. The toxicity of the compounds and their influence on the physical properties of erythrocytes and their membranes as well as model membranes were assessed. Moreover, the mechanism of their interaction with holo-transferrin was determined. To the best of our knowledge, studies on the biological activity of the flavanone methylglucosides have not been performed so far.

## 2. Materials

All reagents were of analytical grade and purchased from Sigma-Aldrich Co., St. Louis, MO, USA. The fluorescent probes, 6-dodecanoyl-2-dimethylaminonaphthalene (Laurdan), 1,6-diphenyl-1,3,5-hexatriene (DPH) were purchased from Molecular Probes, Eugene, OR, USA. The 1-palmitoyl-2-oleoyl-glycero-3-phosphocholine (POPC) was purchased from Avanti Polar Lipids Inc., Alabaster, AL, USA. The human holo-transferrin (HFT, powder, standard grade) was purchased from Sigma-Aldrich Co.

### 2.1. 6-Methylflavanone Biotransformation Products

**Figure 1:**
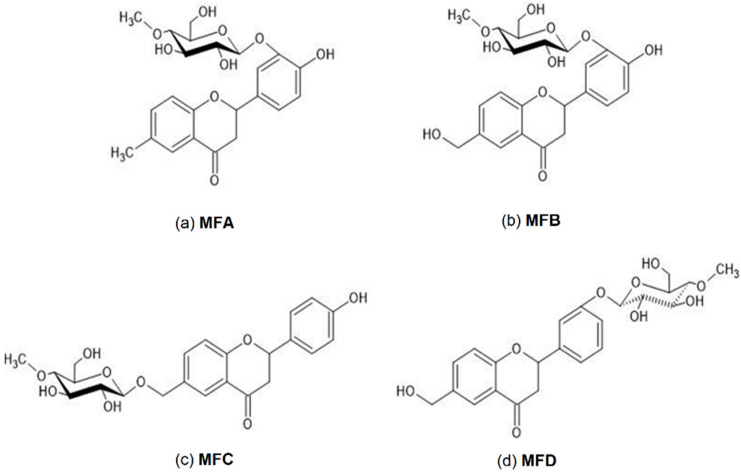
The structures of studied 6-methylflavanone biotransformation products: (**a**) 4′-hydroxy-6-methylflavanone 3′-O-β-D-(4′’-O-methyl)-glucopyranoside (MFA); (**b**) 4′-hydroxy-6-hydroxymethylflavanone 3′-O-β-D-(4′’-O-methyl)-glucopyranoside (MFB); (**c**) 4′-hydroxyflavanone 6-methylene-O-β-D-(4′’-O-methyl)-glucopyranoside (MFC); (**d**) 6-hydroxymethylflavanone 3′-O-β-D-(4′’-O-methyl)-glucopyranoside (MFD).

The products of 6-methylflavanone biotransformation in the cultures of entomopathogenic filamentous fungi were obtained at the Department of Food Chemistry and Biocatalysis, the Wrocław University of Environmental and Life Sciences and previously described [20]. The structures of the tested compounds are presented at Figure 1.

The compounds were dissolved in DMSO at 10 mM concentration and stored in the refrigerator.

### 2.2. Biological and Lipid Membranes

The studies were conducted on isolated erythrocyte membranes, which were obtained from fresh blood using the Dodge et al. (1963) method [21]. The content of erythrocyte membranes in the samples was determined on the basis of protein concentration, which was assayed using the Lowry method [22], and it was 100 µg/mL. The choice of pig erythrocytes was dictated by the fact that this cell’s percentage share of lipids is closest to that of the human erythrocytes, and the blood was easily available. The blood was obtained in the process of slaughtering of slaughter pigs. Fresh blood was taken each time to a physiological solution of sodium chloride with heparin added. The lipids were extracted from erythrocyte membranes according to the method described by Maddy et al. [23].

Large unilamellar liposomes (LUVs) were composed of POPC and lipids extracted from erythrocytes (RBCL). The lipids were dissolved in a chloroform/methanol solvent and evaporated to dryness under nitrogen. Subsequently, a phosphate buffer of pH 7.4 was added and multilamellar vesicles (MLVs) were formed by mechanical shaking. Then MLVs were then 100 times extruded through filters with 100 nm pores (Whatman^®^ GE Healthcare, Chicago, IL, USA), which allowed obtaining vesicles with a mean diameter of 110–120 nm (0.07–0.12 PDI). The fluorescence probes and studied compounds were added to the vesicle solution after their formation.

## 3. Methods

### 3.1. Fluorimetric Studies of Interaction of the Compounds with Biological and Lipid Membrane

The erythrocyte membranes and liposomes were suspended in an isotonic phosphate solution of pH 7.4. In the case of erythrocyte membranes, the protein concentration in the samples amounted to approximately 0.1 mg/mL. The concentration of the lipids (POPC or RBCL) was 250 µM. The control samples contained an erythrocyte ghost or liposome suspension, a fluorescent probe (100:1; lipids/fluorescence probe molar ratio), and an appropriate amount of DMSO. The investigated samples additionally contained appropriate concentrations of the compounds studied dissolved in DMSO. Fluorescence intensity was measured by using two fluorescent probes: Laurdan and DPH, whose concentration in the samples was 2.5 µM, while concentrations of the compounds were within the range of 5–30 µM. The measurements were carried out at a temperature of 37 °C and were conducted with a fluorimeter (CARRY Eclipse of VARIAN, Mulgrave, Australia) equipped with a Peltier temperature controller DBS (Mulgrave, Australia) (temperature accuracy ± 0.1 °C). The excitation and emission wavelengths for the DPH probe were: λ_ex_ = 360 nm, λ_em_ = 425 nm. The excitation wavelength for Laurdan was 360 nm, and the emitted fluorescence was recorded at two wavelengths, 440 and 490 nm. The excitation and emission slits were 5 nm and 10 nm, respectively.

Fluorescence anisotropy (A) for the DPH probe was calculated using the formula [24]:

(1) $$A=\frac{\left(I_{II}-GI_{⊥}\right)}{\left(I_{II}+2GI_{⊥}\right)}$$

where I_II_ and I_⊥_ are fluorescence intensities observed in directions parallel and perpendicular, respectively, to the polarization direction of the exciting wave. G is an apparatus constant dependent on the emission wavelength.

Changes in the polar group packing arrangement of the hydrophilic part of the membrane were investigated using the Laurdan probe, on the basis of its generalized polarization (GP), and were calculated with the formula [24,25]:

(2) $$GP=\frac{\left(I_{b}-I_{r}\right)}{\left(I_{b}+I_{r}\right)}$$

where I_b_ is fluorescence intensity at λ = 440 nm, and I_r_ is fluorescence intensity at λ = 490 nm.

### 3.2. Hemolytic Studies of Toxicity of the Compounds and Their Influence on Osmotic Resistance

The hemolytic experiments were conducted on fresh, heparinized pig blood. Erythrocytes were washed in an isotonic phosphate solution of pH 7.4 and resuspended in this solution for the experiments. Upon removing plasma, the erythrocytes were washed four times in the phosphate solution and then incubated in the same solution but containing appropriate amounts of the compound studied. The modification was conducted at 37 °C for 1 h, each sample containing 10 mL of erythrocyte suspension of 1.2% hematocrit, stirred continuously. After modification, 1 mL samples were taken, centrifuged, and the supernatant was assayed for hemoglobin content using a spectrophotometer (Specord 40, Analytik Jena, Jena, Germany) at 540 nm wavelength. Hemoglobin concentration in the supernatant, expressed as a percentage of hemoglobin concentration in the supernatant of totally hemolyzed cells, was assumed as the measure of the extent of hemolysis.

In the osmotic resistance assay, blood was centrifuged for 3 min, 2500 rev/min at 4 °C to remove the plasma and leukocytes. The erythrocytes obtained were washed four times with a cool (ca. 4 °C) 310 mOsm PBS (phosphate buffered saline, pH 7.4) isotonic solution. Next, a 1.2% RBCs suspension containing compounds of 25 and 50 µM concentrations was prepared and left for 1 h at 37 °C with continuous stirring. After this modification, the suspension of RBCs was centrifuged for 15 min at room temperature in order to remove the cells from the compound’s solution. From the cell sediment, 100 µL samples of the compound-modified cells were taken and suspended in test tubes containing NaCl solutions of 0.5–0.9%. In solutions of the same concentrations were also suspended red blood cells treated with the proper amount of DMSO that constituted the control for osmotic resistance determinations. Then, the suspension was stirred and centrifuged under the above-stated conditions. After that, the percentage of hemolysis was measured with a spectrophotometer at λ = 540 nm wavelength. On the basis of the results obtained, the relation between the percentage of hemolysis and NaCl concentration in the solution was determined. Next, using the obtained hemolytic curves, the impact of the compounds on the osmotic resistance of RBCs was determined. If a hemolytic curve is shifted towards lower NaCl concentration (to the left) the osmotic resistance of the erythrocytes is higher than that of control cells, and vice versa.

### 3.3. Microscopic Studies of the Influence of Compounds on the Shape of Erythrocytes

The RBCs on separation from plasma were washed four times in 0.9% NaCl and suspended in the same solution but containing a proper amount of the compounds studied. Hematocrit of the erythrocytes in the modification solution was 1.2%, the modification lasting 1 h at 37 °C. After modification, the erythrocytes were fixed with a 0.2% solution of glutaraldehyde. After that, the RBCs were observed under a biological optical microscope Nikon Eclipse E200 (Melville, NY, USA) equipped with a digital camera. The obtained photographs made it possible to count erythrocytes of various shapes, and then the percent share of discocytes and different forms of echinocytes and stomatocytes in the population of ca. 500 cells was determined. The concentration of MFA, MFB, MFC, and MFD in this experiment was 25 µM. To the control cells, a proper amount of DMSO was added.

### 3.4. Fluorescence Study of the Holo-Transferrin-Compounds Interactions

The human holo-transferrin (HTF) solution was prepared at a concentration of 4 µM in a phosphate buffer of pH 7.4. The HTF sample concentration was determined spectrophotometrically and was calculated on the expression of the Beer-Lambert law. The molar excitation coefficient of the glycoprotein was assumed as 8.4 × 10^4^ M^−1^cm^−1^. The UV–Vis spectra were recorded using a spectrophotometer (Specord 40, Analytik Jena). The measurements were carried out with a 1 cm × 1 cm quartz cuvette at 23 °C. The stock solutions of the studied compounds were prepared in DMSO at a concentration of 5 mM. HTF solution (2.1 mL, 4 μM) was successively titrated with the increasing concentration of compounds. After addition of the compounds to the HTF solution, the samples were vortexed and incubated for 5 min before measurement. The titrations were completed manually using a micropipette and the concentration of flavonoids was set ranging from 0 to 50 μM. The buffer solution was also read as the background and subtracted to correct the fluorescence spectra. Fluorescence spectra were recorded at 23 °C with the fluorimeter (CARRY Eclipse of VARIAN). The HTF was excited at 295 nm and the emission spectra were collected in the range of 310–500 nm with a step of 1 nm. The excitation and emission slits were 5 nm and 10 nm, respectively. The average of three scans was subjected to smoothing using a 5-point smoothing average. A smoothing step of the data was required to reduce the noise in the spectrum, but the overall shape and intensity of the row emission scan were not affected by the smoothing.

### 3.5. Statistical Analysis

Statistical analysis of the results was performed using the STATISTICA 12.0 (StatSoft PL, Kraków, Poland) software. Statistical analysis was conducted using the Dunnett test (post-hoc test ANOVA) at significance level α = 0.01 or α = 0.05. All the experiments were performed with at least three replicates, and the results were presented as mean ± standard deviation.

### 3.6. Pharmacokinetics and Drug-Likeness Prediction of the 6-Methylflavanone Derivatives

The evaluation of physicochemical properties of the compounds MFA, MFB, MFC, and MFD was performed using SwissADME (Lausanne, Switzerland): a free web tool to evaluate pharmacokinetics, drug-likeness, and medicinal chemistry friendliness of small molecules based on their structural formulae. The structures of the biotransformation products were built by ACD Chemsketch 2021.2.0 (Toronto, Ontario, Canada) and saved in a.mol format, which can be imported into the SwissADME (Lausanne, Switzerland). The prediction results are shown in the Supplementary Materials: Figures S2–S5.

## 4. Results and Discussion

### 4.1. Fluorimetric Studies

The incorporation of flavonoids into the lipid bilayer is sometimes the first step in the sequence of events induced by polyphenolic compounds. Therefore, it is very important to determine their influence on the physical parameters of the membranes, which in turn allows determining the molecular mechanism responsible for their interaction. In order to determine the impact of 6-methylflavanone derivatives on the physical properties of biological and lipid membranes, fluorimetric research was carried out with probes that become embedded at various depths in membranes. In this study, the erythrocyte membranes (ghosts) and liposomes formed from RBCL and POPC were used. Erythrocyte membranes, owing to the presence of functionally relevant membrane protein components embedded in the lipid bilayer, provide a more realistic system for exploring compound actions in biological membranes than simpler membrane models such as POPC. On the other hand, the interpretation of data is much more complicated; therefore, the use of both simple lipid and biological membranes allows for a more detailed study of the interaction of compounds with the membrane. By using the Laurdan probe that emits fluorescence from the hydrophilic area of the membrane [25], the effect of compounds on the degree of order of the polar heads of lipids was investigated. It was established on the basis of changes in the generalized polarization (GP) of the Laurdan probe. Changes in membrane fluidity caused by used compounds were determined on the basis of fluorescence anisotropy (A) of the DPH probe, which becomes located in the area of the hydrocarbon chains of lipids [24].

**Figure 2:**
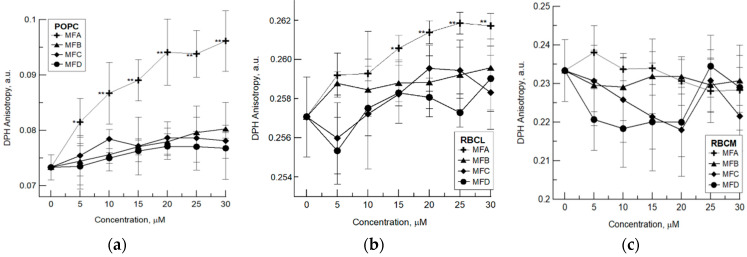
The changes in anisotropy of DPH probe observed under the influence of MFA, MFB, MFC, and MFD compounds in liposomes formed from: (**a**) 1-palmitoyl-2-oleoyl-glycero-3-phosphocholine (POPC); (**b**) lipids extracted from erythrocyte membrane (RBCL); (**c**) in red blood cell membrane (RBCM). The experiment was carried out in three replicates, the results were presented as mean ± standard deviation. Statistically significant differences between modified and control membranes are marked: * α = 0.05; ** α = 0.01.

The obtained results have shown that used compounds have different abilities to change the physical properties of biological and lipid membranes. In relation to the single-component POPC membrane, only the MFA compound significantly changes the membrane’s fluidity, causing an increase in DPH anisotropy (Figure 2a). Similarly, studies in relation to membranes formed from RBCL have shown a slight increase in the DPH anisotropy only in the presence of the MFA compound (Figure 2b). This means that MFA at the used concentration range decreases the fluidity of the lipid membrane. Fluorimetric and FTiR studies have shown that 4′-methylflavone also caused ordering of the hydrophobic part of the lipid membrane composed of phosphatidylcholine [26].

On the other hand, studies in relation to protein–lipid erythrocyte membrane showed a lack of changes in membrane fluidity induced by all studied compounds (Figure 2c). It means that used compounds do not modify the hydrophobic area of the erythrocyte membrane in the used concentration range. The lack of changes induced by the compounds in the fluidity of the RBC membrane is due to the fact that the molecular interactions in the erythrocyte membrane are more complex. The presence of glycocalyx and surface charge may hinder the interaction of these compounds with the membrane.

**Figure 3:**
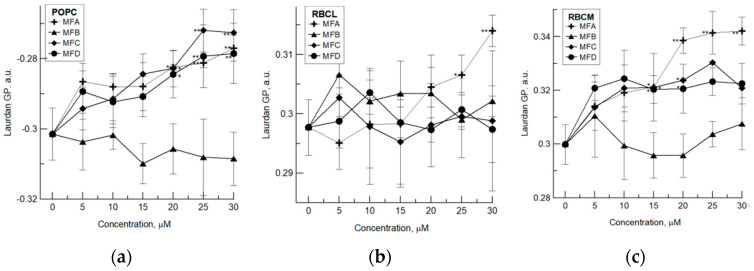
The changes in generalized polarization (GP) of Laurdan probe observed under the influence of MFA, MFB, MFC, and MFD compounds in: (**a**) 1-palmitoyl-2-oleoyl-glycero-3-phosphocholine (POPC) liposomes; (**b**) liposomes formed from lipids extracted from erythrocyte membrane (RBCL); (**c**) red blood cell membrane (RBCM). The experiment was carried out in three replicates, the results were presented as mean ± standard deviation. Statistically significant differences between modified and control membranes are marked: * α = 0.05; ** α = 0.01.

The studies using the Laurdan probe have shown that MFA, MFC, and MFD compounds at concentrations of 15 µM and higher increase values of generalized polarization (GP) of this probe attached to the POPC membrane (Figure 3a). There was no statistically significant difference between the GP changes induced by these compounds. Observed GP changes indicate that the area of the polar heads of lipids is more ordered. The restriction of the movement of the polar heads of lipids is probably a result of the binding of these compounds in this area. The studies using RBCL LUVs showed that only MFA, used at concentrations of 25 and 30 µM, increases the value of GP, causing a slight increase in the degree of order in the hydrophilic region of the membrane (Figure 3b). These results indicate that MFA has the greatest ability to modify different lipid membranes among the tested compounds. Moreover, in the case of MFA, the increase in fluidity both in POPC and RBCL membranes at the hydrophobic region, and the increase in packing order in the hydrophilic part, indicate that the binding of this compound to the lipid membrane is stronger than the other studied compounds. The observed ability of MFA to bind to the membrane and induct changes in its fluidity is related to higher hydrophobicity of this molecule due to the presence of a methyl group at C-6 of ring A. Literature data indicate that methylation of flavonoids increases the hydrophobicity of the molecule and improves their affinity for the cell membrane in relation to unmethylated analogs [9]. Furthermore, the distribution of flavonoids in the membrane is broad and depends on the polarity of the compounds. For the unpolar compounds, i.e., flavone, it is biased towards the hydrophobic core of the bilayer, and for more polar compounds, i.e., luteolin, towards the aqueous phase. Generally, as the flavonoids become more hydrophilic, their membrane localization is more shifted towards the aqueous environment [27]. In order to determine the lipophilicity of the compounds, the evaluation of their physicochemical properties was made using SwissADME software (Figures S2–S5, Supplementary Materials). It showed that the order of lipophilicity of used compounds, determined on the basis of octanol/water partition coefficient (log P) is as follows: MFA > MFC ≥ MFD > MFB. Therefore, the MFA may penetrate the lipid membrane deeper than the other tested compounds, but due to the presence of a sugar residue, it is probably located in the glycerol/head group region of the membrane lipids. And the changes induced in the hydrophobic interior of the membrane are an indirect effect of MFA interaction with the lipid–water interface. The MFC and MFD compounds are more hydrophilic due to the presence of hydroxyl groups and a sugar residue; therefore, they remain in the head group region of the membrane, where they may interact electrostatically and via hydrogen bonds. The MFB is the only molecule with two OH-groups (apart from the glucoside), and these two groups are on different parts of the molecule. Therefore, the MFB structure and high polarity influence their ability to interact with the lipid membrane, due to different physical interactions in the complex physical environment of a lipid membrane. MFB showed no effect in the Laurdan experiments due to less membrane binding; thus, it is mainly located at the surface of the membrane.

Furthermore, the interaction of MFA, MFC, and MFD compounds with the RBC protein–lipid membrane results in growth in the packing order of its hydrophilic region (Figure 3c). This increase confirms the binding of these compounds to the area of the polar heads of lipids. As in previous studies, the largest ordering effect was observed for the MFA compound. Thus, this indicates that MFA has the greatest potential to modify the physical properties of both the protein–lipid and the lipid membrane. Due to the presence of the methyl group, this compound probably interacts not only with the lipid phase but also with proteins of the erythrocyte membrane. The FTiR analysis of amino I and amino II bands of red blood cell membrane protein demonstrated that 4′-methylflavanone and 4′-methylflavone are able to affect the structure of proteins present in this membrane [26].

All above-mentioned changes in the physical properties of the lipid and protein–lipid membranes suggest that used compounds are located mainly in the hydrophilic region of the membrane, whereas the changes in the area of hydrocarbon chains of lipids are probably the result of the modification in polar heads mobility and spatial orientation and/or in the case of erythrocyte, membrane interaction with membrane proteins after the binding of the tested compounds on the membrane’s surface. These compounds, like other glycosylated derivatives of flavanones, can form a hydrogen bond with the polar heads of lipids at the lipid–water interface. Naringenine-7-rhamnosidoglucoside orders the hydrophobic region of the DMPC membrane and induces alterations in the arrangement of polar heads by the formation of strong hydrogen bonding with PO_2_^−^ moieties of lipids [28]. The presence of sugar moiety increases the hydrophilicity of the compound and its size, changes the spatial structure of the molecules, and in consequence, limits their penetration into the deeper areas of the membrane [6,28].

### 4.2. Hemolytic Studies

The ability of the MFA, MFB, MFC, and MFD compounds to induce damage in the red blood cells was determined in hemolytic studies based on hemoglobin concentration released from cells modified by these compounds. After one hour of RBC modification with the compounds used at concentrations from 5 to 50 µM, no increased hemolysis was observed as compared to the cells treated with the same amount of DMSO alone (Figure S1, Supplementary Materials). This means that the compounds used show no RBC toxicity over the used concentration range. As in our studies, the lack of toxic effect of, i.e., kaempferol, quercetin, morin, rutin, and cyanidin glycosides, on erythrocytes was shown in [29,30]. Additionally, these compounds protect RBCs against oxidative hemolysis induced by tetrathionate and AAPH.

**Figure 4:**
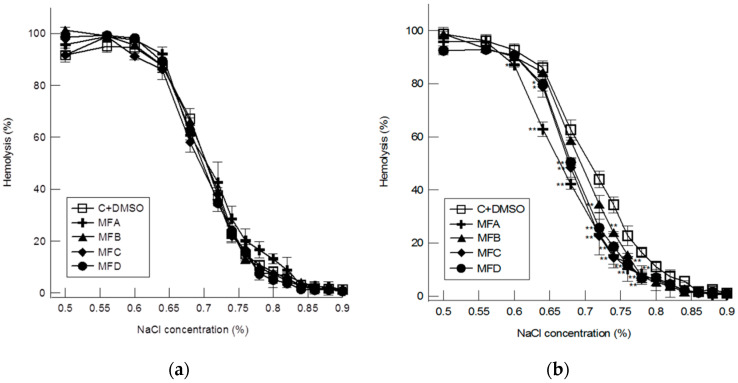
The relationship between the percentage of hemolysis of control and compounds modified RBCs vs. the percentage of sodium chloride (NaCl). The compounds were used at 25 µM (**a**) and 50 µM (**b**) of concentrations. The experiment was carried out in three replicates, the results were presented as mean ± standard deviation. Statistically significant differences are marked: * α = 0.05; ** α = 0.01.

The impact of the compounds on RBC membrane osmotic resistance was tested at concentrations of 25 µM and 50 µM. To control RBCs, the same amount of DMSO as in the compound-modified cells was added. On the basis of the obtained results, the hemolytic curves were plotted, which show the dependence of the percentage of RBC hemolysis vs. the percentage concentration of NaCl (Figure 4). At concentrations of 25 µM, the MFA, MFB, MFC, and MFD compounds do not significantly impact the RBC stability against hypotonic lysis. This is in accordance with the results of fluorimetric studies that showed no changes in RBCs membrane fluidity and only a slight ordering effect in the area of the polar head of lipids to the compound concentration of 30 µM. At a doubly higher concentration (50 µM) a shift of the osmotic resistance curve towards lower concentrations of NaCl was observed. The first significant decrease in the degree of hemolysis of RBC modified with the tested compounds was found for the NaCl concentration of 0.77%. As the concentration of NaCl decreased, the differences between the control and modified cells increased up to 0.6% of NaCl. The reduced hemolysis of modified RBCs as compared to control cells, at the same concentration of NaCl, indicates their greater resistance to changes in osmotic pressure. Furthermore, comparison of the changes induced by the compounds shows that MFA has the greatest ability to modify the osmotic resistance of RBCs which is consistent with the results of fluorimetric studies. The magnitude of the changes induced by MFC and MFD is comparable but lower than induced by MFA and also slightly greater than that caused by MFB. Weak interaction between MFB and RBCs confirms the results of fluorimetric tests, which showed no membrane-modifying activity of MFB to the concentration of 30 µM.

### 4.3. Microscopic Studies

**Figure 5:**
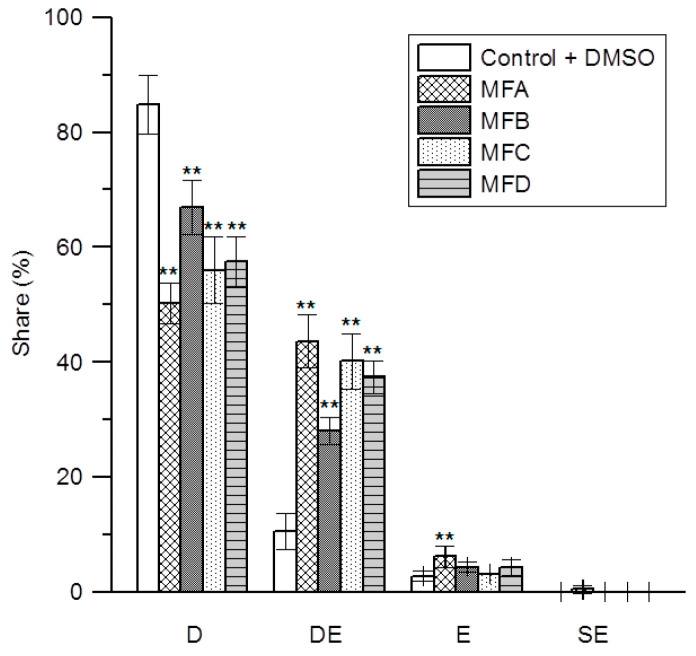
The percentage share of discocytes (D), discoechinocytes (DE), echinocytes (E), and spheroechinocytes (SE) in population of control erythrocytes (C + DMSO) and those modified by MFA, MFB, MFC, and MFD compounds used at 25 µM concertation. The experiment was carried out in three replicates, and the results were presented as mean ± standard deviation. Statistically significant differences are marked: ** α = 0.01.

The hemolytic studies showed no destructive effect of the tested compounds on erythrocytes, but even enhanced stability of erythrocytes against hypotonic lysis. Therefore, in order to confirm the location of these compounds in the erythrocyte membrane, their influence on the shape of RBCs was investigated using an optical microscope. Control and modified cells were photographed, and on the basis of the obtained photos, the percentage share of individual forms of erythrocytes in the population of at least 500 cells was calculated. Individual forms of RBCs were identified according to Bessis and Brecher’s scale [31]. The cells were therefore assigned to the following groups: disocytes (D), spherocytes (SS), stomatocytes (S), discostomatocytes (DS), discoechinocytes (DE), echinocytes (E), spheroechinocytes (SE), and spherocytes (ES) resulting from the transformation of spheroechniocytes. The obtained results showed that MFA, MFB, MFC, and MFD compounds used at 25 µM concentration induced the transformation of the part of discocytes (D) mainly into discoechinocytes (DE) (Figure 5). Furthermore, in the case of MFA-modified cells, a statistically important increase in the number of echninocytes (E) relative to the control cells was observed. The total share of other forms of erythrocytes (SS, S, DS, and ES) in all the samples was negligible and did not exceed 2%. There are also no statistically significant differences between the activity of MFA in relation to MFC, and MFD, but the obtained results indicate a significantly higher activity of MFA compared to MFB. The MFC and MFD shape-changing activity is also slightly higher than that of MFB. According to the Sheetz and Singer theory [32], the compounds located in the outer lipid layer of the membrane induce the formation of echinocytes, whereas those penetrating the inner monolayer induce the formation of stomatocytes. The results obtained in the microscopic investigation are in accord with the results of fluorimetric studies and prove that the used 6-methylflavanone derivatives permeate the hydrophilic lipid layer of the erythrocyte membrane. The transformation of discocytes into echinocytes under the influence of flavonoids and their glycosides was earlier documented, i.e., for cyanidin and its glycosides [33].

On the basis of all obtained results, it can be concluded that the tested compounds are located in the hydrophilic region of the lipid membrane. However, their ability to modify the physical properties of membranes is strictly dependent on the number, type, and location of the substituent in the structure of flavanone and also on the type of the membrane.

### 4.4. Interaction of the Compounds with HTF

In order to determine the compound–HTF interaction, the fluorescence quenching method was used. This method provides insight into the binding mechanism, driving forces, binding constant, intermolecular distances between ligands and proteins, and also the microenvironment of the chromophore groups in proteins [34]. A decrease in the quantum yield of fluorescence from a fluorophore is due to a variety of molecular interactions, and a mechanism of this quenching is usually classified as either dynamic or static quenching. The static quenching takes place when the fluorophore–quencher complex is formed, whilst the dynamic one refers to a process that involves the fluorophore and the quencher molecule coming into contact during the transient existence of the excited state.

**Figure 6:**
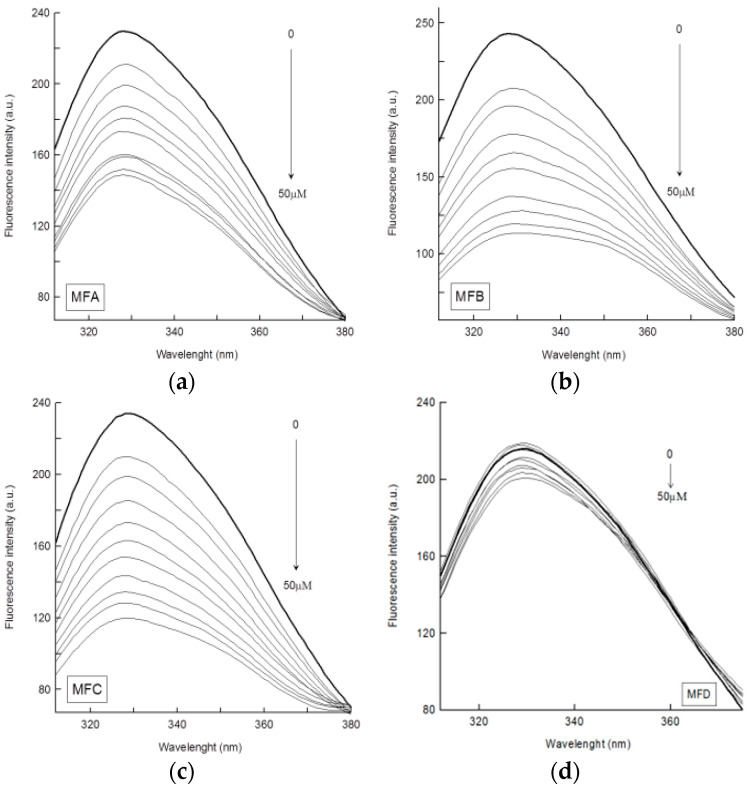
Fluorescence emission spectra of the (**a**) MFA-HTF system, (**b**) MFB-HTF system, (**c**) MFC-HTF system, and (**d**) MFD-HTF system. The concentration of HTF was 4 µM and that of the compounds was increased from 5 to 50 µM with a step of 5 µM (λ_ex_ = 295 nm, T = 23 °C). The arrow direction shows an increase in compound concentration.

**Figure 7:**
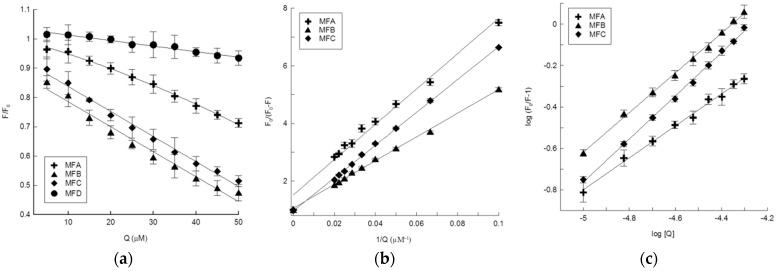
Quenching curves of holo-transferrin (HTF) in the presence of MFA, MFB, MFC, and MFD compounds used in the concentration range from 0 to 50 µM. (**a**) Relative fluorescence intensity (F/F_0_) was calculated as the ratio of the HTF fluorescence intensity in the presence of compound (F) to the HFT fluorescence intensity before compound addition (F_0_). (**b**) Stern–Volmer curves of HTS-compounds complex (**c**) logarithmic plots of HTF fluorescence quenching. The HTF was excited at 295 nm, the measurements were done at 23 °C. The results are presented as an average ± standard deviation.

The intrinsic fluorescence of HTF is due to 8 tryptophan and 26 tyrosine residues. When HTF is excited at 280 nm, the fluorescence comes from both tryptophan and tyrosine residues. In this study, HTR was excited at 295 nm, and the observed fluorescence emission originates only from its tryptophan residues [34]. The fluorescence spectra of HTF in phosphate buffer (pH 7.4) in the absence and presence of 6-methylflavone biotransformation products showed strong fluorescence emission with a peak at 328 nm (Figure 6). Under the same conditions, the compounds alone exhibited much weaker fluorescence emission in the range of 380–480 nm. The ratio of the absolute molar absorption coefficient of HTF to that of compounds at 295 nm was more than 12 for MFC (HTF/MFC) and MFD (HTF/MFD) and more than 24 for MFA (HTF/MFA) and MFB (HTF/MFB); thus, the absorption of the used compounds had a negligible effect on HTF fluorescence intensity throughout the entire titration experiment. After the addition of MFA, MFC, and MFB to HTF solutions, protein fluorescence intensity decreases with the increasing concentration of the compounds (Figure 6a–c). MFD practically does not quench the protein’s fluorescence because of the slight decrease in fluorescence intensity (less than 8% at the highest concentration of 50 µM) under the influence of this compound (Figure 6d). The observed slight decrease in fluorescence intensity is within the error of the method and is the effect of small compounds absorption and protein dilution in the used titration method rather than MFD-HTF interaction. The remaining tested compounds quench HTF fluorescence to a varying extent. The comparison of changes in HTF relative fluorescence intensity (F/F_0_) under the influence of the tested compounds (Figure 7) indicates that the quenching effect was stronger for MFB and MFC than for the MFA compound.

In order to determine the HTF fluorescence quenching mechanism by the tested compounds, a Stern–Volmer analysis was applied (Equation (3)) [35]:

(3) $$\frac{F_{0}}{F_{0}-F}=\frac{1}{f_{a}K_{SV}\left[Q\right]}+\frac{1}{f_{a}}$$

where F_0_—fluorescence intensity of HTF, F—fluorescence intensity of HTF in the presence of the tested compounds, Q—quencher concentration, K_SV_—Stern–Volmer quenching constant, f_a_—fraction of fluorophore accessible to the quencher.

For the used compounds, the Stern–Volmer curves (F_0_/(F_0_ − F) vs. 1/[Q]) are linear over the entire used concentration range (Figure 7b). This indicates that only one class of fluorophores exists in the protein and that there is only one dynamic or static quenching mechanism. From the intercept and slope in the Stern–Volmer curves, the parameters f_a_ and K_SV_ were calculated. The quenching rate constant of the biomolecule K_q_ was calculated as a ratio of the Stern–Volmer quenching constant (K_SV_) and the average lifetime of HST (τ_0_) in the absence of the quencher. The value of τ_0_ was found to be 2.5 ns [35]. The calculation results are listed in Table 1. The values of the Stern–Volmer quenching constants are of the same order of magnitude as those obtained for flavanone naringenin (9.55 × 10^4^ M^−1^) with the same skeleton structure [33]. The calculated K_q_ values for MFA, MFB, and MFC are in the order of 10^12^ M^−^^1^ s^−^^1^ and the minimum was 6.8 × 10^12^ M^−^^1^ s^−^^1^ obtained for MFC (Table 1). These values are much higher than the maximum scatter collision quenching constant of dynamic quenching (2 × 10^10^ M^−^^1^ s^−^^1^), which suggests that there is a specific interaction between HTF and the 6-methylflavanone glucosides and indicates that the static quenching mechanism occurred. The literature data also showed that the quenching mechanism between different flavonoids and HTS is static and occurs via complex formation [34,36,37]. The same type of interaction was also observed between bovine serum albumin (BSA) and various flavonoids [35,38] studied by fluorescence quenching methods.

In the case of static quenching presses, the binding constant (K_a_) and the number of binding sites (n) may be calculated from the regression curve based on the Scatchard equation [34]:

(4) $$\mathrm{lo}\left(\frac{F_{0}}{F}-1\right)={\;\mathrm{logK}}_{a}+n\;\log\;\left[Q\right]$$

where F_0_—fluorescence intensity of HTF, F—fluorescence intensity of HTF in the presence of the tested compounds, K_a_—binding constant, Q—concentration of the quencher, n—binding sites per HTF molecule.

The binding constants (K_a_) and a number of binding sides (n) for MFA, MFB, and MFC compounds were obtained from the plots of log (F_0_/F − 1) vs. log[Q] as a slope and intercept on the *y*-axis, respectively (Figure 7c). The plots for all compounds showed the linear behavior and the calculated parameters are listed in Table 1. The binding constant values obtained for MFA, MFB, and MFC compounds are about 3–4 times smaller than values published for other flavonoids, for example: apigenin (6.7 × 10^4^ M^−1^), flavanone (6.9 × 10^4^ M^−1^), 3-hydroxyflavone (7.6 × 10^4^ M^−1^), and luteolin (8.2 × 10^4^ M^−1^) [34,36,37]. It is considered that the binding ability generally increases with the increasing number of hydroxyl groups in flavonoid molecules [37]. The tested compounds, except for the methylglucose unit, possess in their structure one hydroxyl group attached to ring B (MFA, MFB, MFC) and/or one hydroxymethyl group attached to ring A (MFB, MFD), which explains their lower binding capacity with respect to the above-mentioned flavonoids. The K_a_ value determined for MFA is lower than that determined for MFB, which confirms the dependence of the binding ability on the number of hydroxyl groups. Apart from the number of hydroxyl groups, their position in the structure is also of great importance. Our results indicate that the hydroxyl groups in ring B may determine the interaction of *O*-methylglucosides of flavanones with transferrin. Like MFB, MFD has one hydroxymethyl group at position C-6 of ring A obtained by oxidation of the methyl group, and also the methylglucose unit at C-3′ of ring B. The MFD compound does not interact with HTF probably due to the absence of the hydroxyl group attached to ring B. Furthermore, the binding ability of the tested compounds is also 100 times lower than that determined for naringenin (6.3 × 10^6^ M^−1^) [34], the aglycone that possesses the same skeleton structure with three hydroxyl groups (4′ at ring B and 5,7 at ring A), which indicates that the presence of a methylglucose moiety significantly reduces their interaction with HTF. As in our study, literature data indicate that glycosylation had an obvious influence on the binding affinity of different flavonoids on the BSA. For flavanones, the introduction of the glucose unit may increase the molecular size of flavonoid structure and then prevent these flavonoids from accessing the active center of BSA [38]. Additionally, when considering the effect of MFA, MFB, and MFC on the fluorescence spectra of HTF, there was no apparent shift in the emission maximum (λ_em_). This suggests that there are no other changes in the immediate environment of the tryptophan residues except the fact that the compounds are situated in close proximity to the tryptophan residue for the quenching effect to occur. Moreover, the lack of λ_em_ shift may also indicate that the molecular conformation of the protein was not affected due to their influence [35]. As with other flavonoids, noncovalent bonding such as hydrogen bonding, ionic, and hydrophobic interaction are likely to be important driving forces for protein–flavanone *O*-methylglucoside association [35,37].

## 5. Conclusions

The used flavanone *O*-methylglucosides to varying degrees induce changes in the hydrophobic and hydrophilic areas of lipids and biological membranes. The greatest membrane-modifying ability was observed for the compound with a methyl group at C-6 of ring A, which is responsible for the increased hydrophobicity of this compound compared to other tested compounds. Additionally, the used compounds are nontoxic for erythrocytes. Their binding to the RBCs results in a modification of their shape and makes the RBC membrane more resistant to pressure changes in the surrounding environment. Furthermore, the tested compounds are able to form complexes with holo-transferrin. They, like other flavonoids, bind to the protein noncovalently and do not change the polarity of the microenvironment of tryptophan residues. Their interaction with human holo-transferrin depends on the number and the position of the hydroxyl groups at the skeleton structure of the molecule. Generally, the biological activity of the flavanone *O*-methylglucosides used in these studies does not differ significantly from that of other similar glycosylated derivatives of flavonoids. Therefore, it is necessary to conduct additional, more specialized research on their biological activity in order to determine the possibility of their practical use in disease prevention and treatment.

## Figures and Tables

**Table 1:** Biophysical parameters of holo-transferrin (HTF) interactions with 6-methylflavanone biotransformation products were determined from the Stern-Volmer and Scatchard equations at 23 °C. The Stern–Volmer quenching constant (K_SV_), bimolecular quenching constant (K_q_), binding constant (K_a_), and binding sites per protein molecule (n).

System	K_SV_ (M^−1^) × 10^4^	R*	K_q_ (s^−1^ M^−1^) × 10^12^	K_a_ (M^−1^) × 10^4^	R**	n
HTF-MFA	2.50	0.983	9.92	1.12	0.994	0.77
HTF-MFC	1.70	0.998	6.80	2.79	0.998	1.04
HTF-MFB	2.58	0.999	10.3	1.47	0.998	0.96

R* is the correlation coefficient for the K_SV_ values, R** is the correlation coefficient for the K_a_ values.

## Data Availability

Not applicable.

## Supplementary Materials

The following supporting information can be downloaded at: https://www.mdpi.com/article/10.3390/membranes12100978/s1, Figure S1: The percentage of hemolysis of RBC in the absence (C + DMSO) and in the presence of MFA, MFB, MFC, and MFD compounds.; Figures S2–S5: The physicochemical properties of MFA, MFB, MFC, MFD compounds obtained by computational simulation performed with using SwissADME.
