# Supplementary material for: Novel O-Methylglucoside Derivatives of Flavanone in Interaction with Model Membrane and Transferrin
Source: Membranes (Basel). 2022 Oct 8;12(10):978. doi: 10.3390/membranes12100978 (PMC9609356; doi:10.3390/membranes12100978)
Supplement: Supplementary file 1 [file membranes-12-00978-s001.zip › membranes-1952896-supplementary.pdf]

membranes

Supplementary Materials

Novel *O*-Methylglucoside Derivatives of Flavanone in Interaction with Model Membrane and Transferrin

Sylwia Cyboran-Mikołajczyk [1,*], Dorota Bonarska-Kujawa [1], Katarzyna Męczarska [1], Agnieszka Krawczyk-Łebek [2] and Edyta Kostrzewa-Susłow [2]

[1] Department of Physics and Biophysics, Wrocław University of Environmental and Life Sciences, Norwida 25, 50-375 Wrocław, Poland
[2] Department of Food Chemistry and Biocatalysis, Wrocław University of Environmental and Life Sciences, Norwida 25, 50-375 Wrocław, Poland
* Correspondence: sylwia.cyboran@upwr.edu.pl

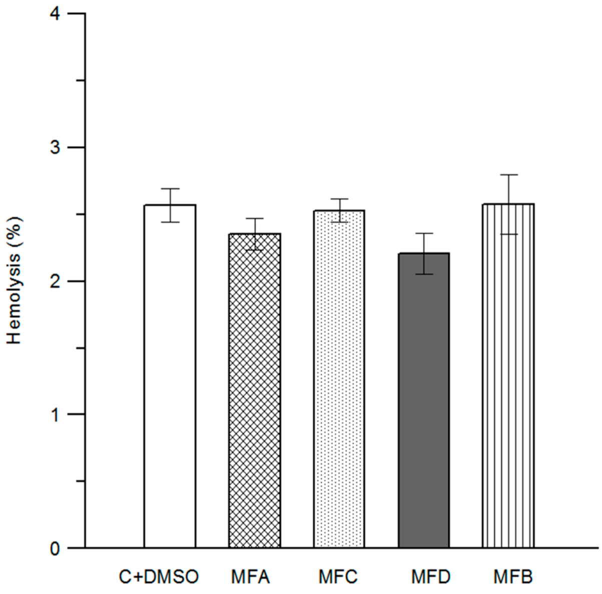

Figure S1. The percentage of hemolysis of RBC in the absence (C + DMSO) and in the presence of MFA, MFB, MFC, and MFD compounds used at 50 µM concentration. The experiment was carried out in five replicates, the results were presented as mean ± standard deviation.

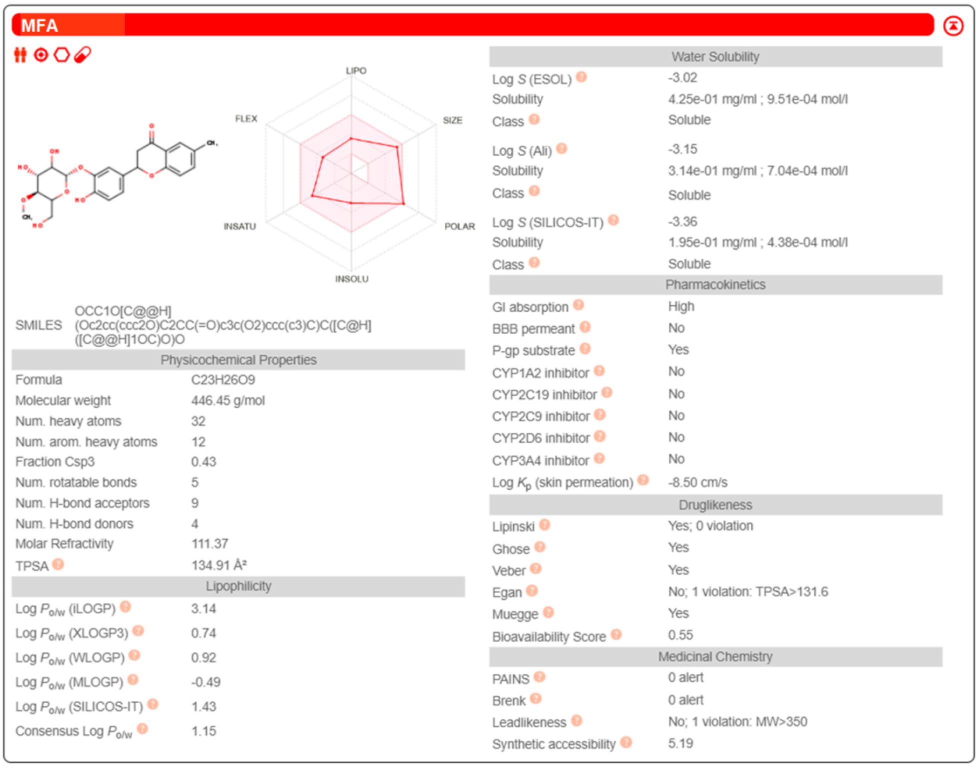

Figure S2. The physicochemical properties of 4′-hydroxy-6-methylflavanone 3′-O-β-D-(4′′-O-methyl)-glucopyranoside (MFA) compound obtained by computational simulation performed with using SwissADME based on the compounds structural formulae.

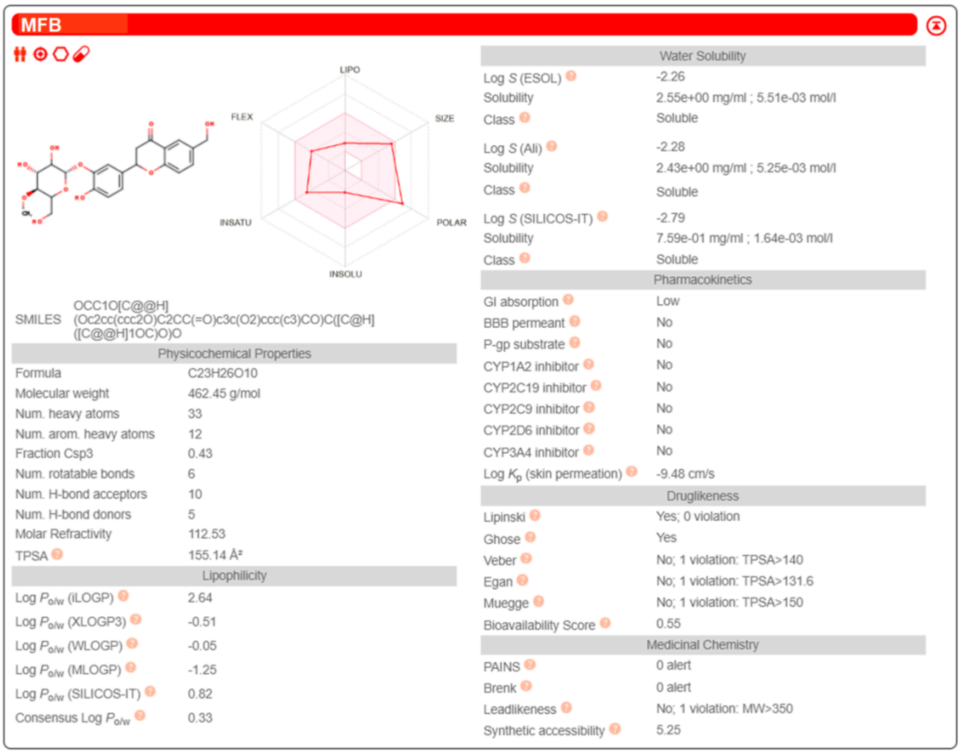

SMILES OCC1O[C@@H](Oc2cc(ccc2O)C2CC(=O)c3c(O2)ccc(c3)CO)C([C@H]([C@@H]1OC)O)O

Physicochemical Properties	
Formula	C23H26O10
Molecular weight	462.45 g/mol
Num. heavy atoms	33
Num. arom. heavy atoms	12
Fraction Csp3	0.43
Num. rotatable bonds	6
Num. H-bond acceptors	10
Num. H-bond donors	5
Molar Refractivity	112.53
TPSA	155.14 Å²

Lipophilicity	
Log $P_{o/w}$ (iLOGP)	2.64
Log $P_{o/w}$ (XLOGP3)	-0.51
Log $P_{o/w}$ (WLOGP)	-0.05
Log $P_{o/w}$ (MLOGP)	-1.25
Log $P_{o/w}$ (SILICOS-IT)	0.82
Consensus Log $P_{o/w}$	0.33

Water Solubility	
Log *S* (ESOL)	-2.26
Solubility	2.55e+00 mg/ml ; 5.51e-03 mol/l
Class	Soluble
Log *S* (Ali)	-2.28
Solubility	2.43e+00 mg/ml ; 5.25e-03 mol/l
Class	Soluble
Log *S* (SILICOS-IT)	-2.79
Solubility	7.59e-01 mg/ml ; 1.64e-03 mol/l
Class	Soluble

Pharmacokinetics	
GI absorption	Low
BBB permeant	No
P-gp substrate	No
CYP1A2 inhibitor	No
CYP2C19 inhibitor	No
CYP2C9 inhibitor	No
CYP2D6 inhibitor	No
CYP3A4 inhibitor	No
Log K_p (skin permeation)	-9.48 cm/s

Druglikeness	
Lipinski	Yes; 0 violation
Ghose	Yes
Veber	No; 1 violation: TPSA>140
Egan	No; 1 violation: TPSA>131.6
Muegge	No; 1 violation: TPSA>150
Bioavailability Score	0.55

Medicinal Chemistry	
PAINS	0 alert
Brenk	0 alert
Leadlikeness	No; 1 violation: MW>350
Synthetic accessibility	5.25

Figure S3. The physicochemical properties of 4′-hydroxy-6-hydroxymethylflavanone 3′-O-β-D-(4′′-O-methyl)-glucopyranoside (MFB) compound obtained by computational simulation performed with using SwissADME based on the compounds structural formulae.

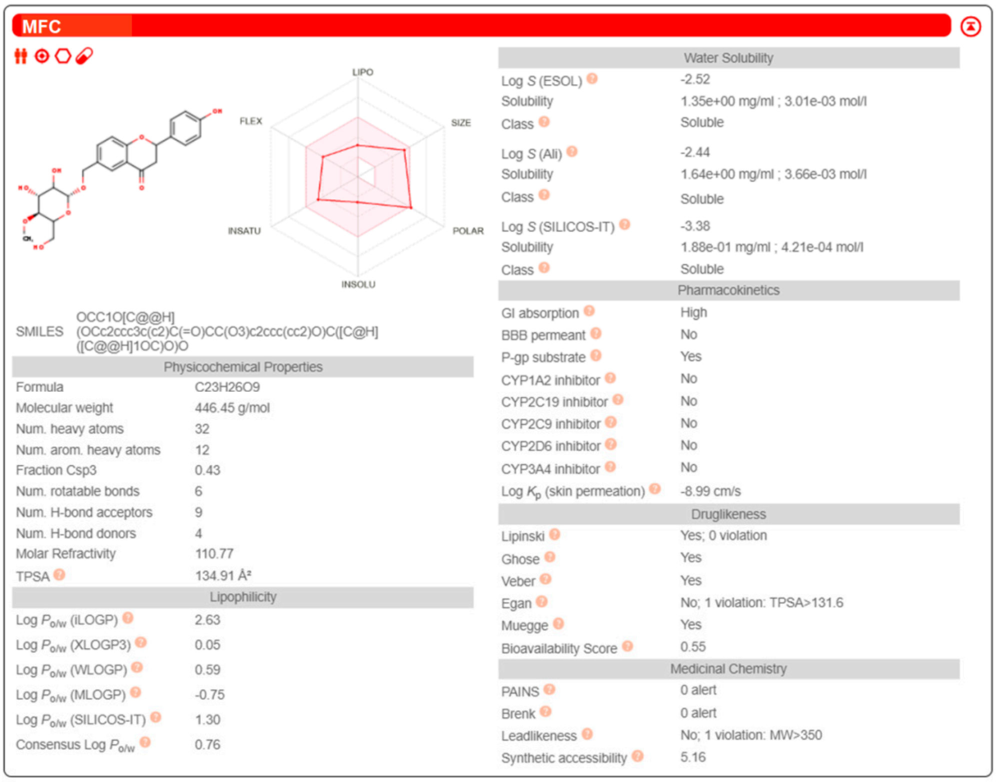

SMILES OCC1O[C@@H](OCc2ccc3c(c2)C(=O)CC(O3)c2ccc(cc2)O)C([C@H]([C@@H]1OC)O)O

Physicochemical Properties	
Formula	C23H26O9
Molecular weight	446.45 g/mol
Num. heavy atoms	32
Num. arom. heavy atoms	12
Fraction Csp3	0.43
Num. rotatable bonds	6
Num. H-bond acceptors	9
Num. H-bond donors	4
Molar Refractivity	110.77
TPSA	134.91 Å²

Lipophilicity	
Log $P_{o/w}$ (iLOGP)	2.63
Log $P_{o/w}$ (XLOGP3)	0.05
Log $P_{o/w}$ (WLOGP)	0.59
Log $P_{o/w}$ (MLOGP)	-0.75
Log $P_{o/w}$ (SILICOS-IT)	1.30
Consensus Log $P_{o/w}$	0.76

Water Solubility	
Log *S* (ESOL)	-2.52
Solubility	1.35e+00 mg/ml ; 3.01e-03 mol/l
Class	Soluble
Log *S* (Ali)	-2.44
Solubility	1.64e+00 mg/ml ; 3.66e-03 mol/l
Class	Soluble
Log *S* (SILICOS-IT)	-3.38
Solubility	1.88e-01 mg/ml ; 4.21e-04 mol/l
Class	Soluble

Pharmacokinetics	
GI absorption	High
BBB permeant	No
P-gp substrate	Yes
CYP1A2 inhibitor	No
CYP2C19 inhibitor	No
CYP2C9 inhibitor	No
CYP2D6 inhibitor	No
CYP3A4 inhibitor	No
Log K_p (skin permeation)	-8.99 cm/s

Druglikeness	
Lipinski	Yes; 0 violation
Ghose	Yes
Veber	Yes
Egan	No; 1 violation: TPSA>131.6
Muegge	Yes
Bioavailability Score	0.55

Medicinal Chemistry	
PAINS	0 alert
Brenk	0 alert
Leadlikeness	No; 1 violation: MW>350
Synthetic accessibility	5.16

Figure S4. The physicochemical properties of 4′-hydroxyflavanone 6-methylene-O-β-D-(4′′-O-methyl)-glucopyranoside (MFC) compound obtained by computational simulation performed with using SwissADME based on the compounds structural formulae.

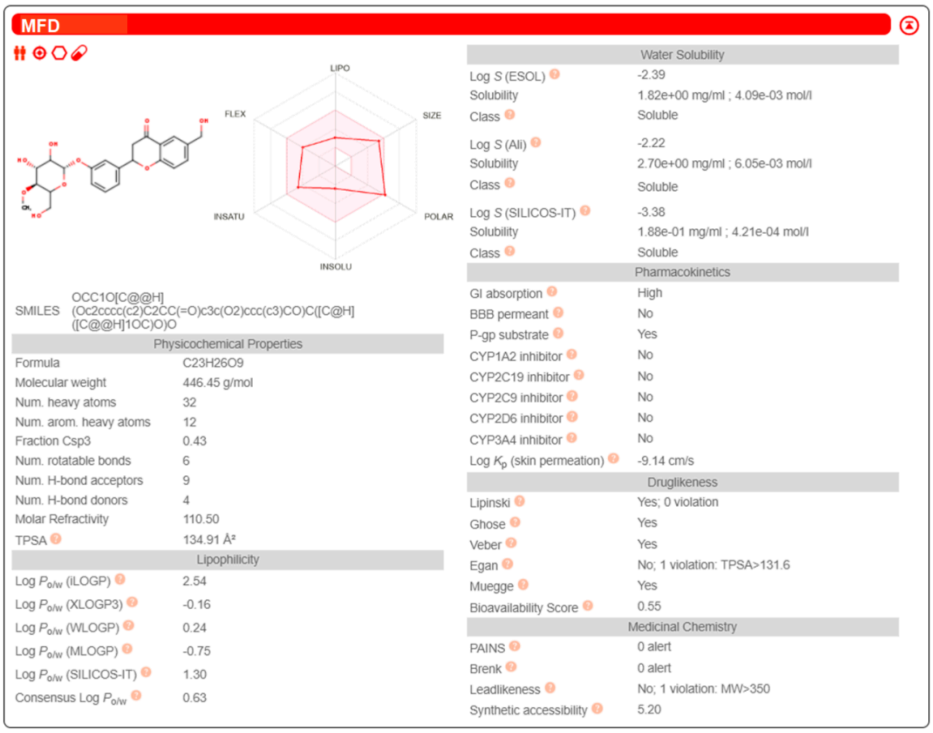

Figure S5. The physicochemical properties of 6-hydroxymethylflavanone 3′-O-β-D-(4′′-O-methyl)-glucopyranoside (MFD) compound obtained by computational simulation performed with using SwissADME based on the compounds structural formulae.
